# P-311. Shifts in Methicillin-Resistant *Staphylococcus aureus* Types in Bloodstream Infections: Nationwide and Regional Influences and Their Impact on Mortality

**DOI:** 10.1093/ofid/ofae631.514

**Published:** 2025-01-29

**Authors:** Norihito Kaku, Masaki Ishige, Go Yasutake, Daisuke Sasaki, Kenji Ota, Fujiko Mitsumoto-Kaseida, Kosuke Kosai, Hiroo Hasegawa, Koichi Izumikawa, Hiroshi Mukae, Katsunori Yanagihara

**Affiliations:** Nagasaki University Hospital, Nagasaki, Nagasaki, Japan; Nagasaki University, Nagasaki, Nagasaki, Japan; Nagasaki University, Nagasaki, Nagasaki, Japan; Nagasaki University Hospital, Nagasaki, Nagasaki, Japan; Nagasaki University, Nagasaki, Nagasaki, Japan; Nagasaki University Graduate School of Biomedical Sciences, Nagasaki, Nagasaki, Japan; Nagasaki University, Nagasaki, Nagasaki, Japan; Nagasaki University, Nagasaki, Nagasaki, Japan; Nagasaki University, Nagasaki, Nagasaki, Japan; Nagasaki University, Nagasaki, Nagasaki, Japan; Nagasaki University, Nagasaki, Nagasaki, Japan

## Abstract

**Background:**

Methicillin-resistant *Staphylococcus aureus* (MRSA) is the leading cause of bloodstream infections (BSI). In the USA and Japan, the New York/Japan clone (ST5-II) was historically prevalent, but recent studies in Japan have shown a shift towards ST8-IV and CC1-IV as the dominant strains. The extent to which these trends are mirrored in individual facilities and the variation in patient backgrounds across different MRSA types remains unclear.

**Figure:**
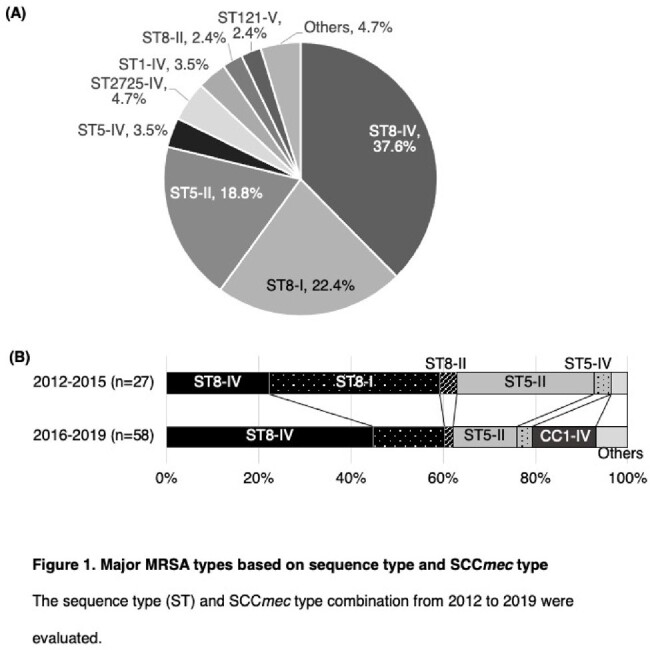

**Methods:**

We analyzed MRSA strains from blood cultures collected at Nagasaki University between 2012 and 2019. Whole-genome sequencing was utilized to determine sequence types, SCCmec, spa types, and resistance and virulence genes, complemented by phylogenetic analysis using core-genome MLST. Data were compared with earlier studies from 2003-2011 at the same hospital.

**Figure:**
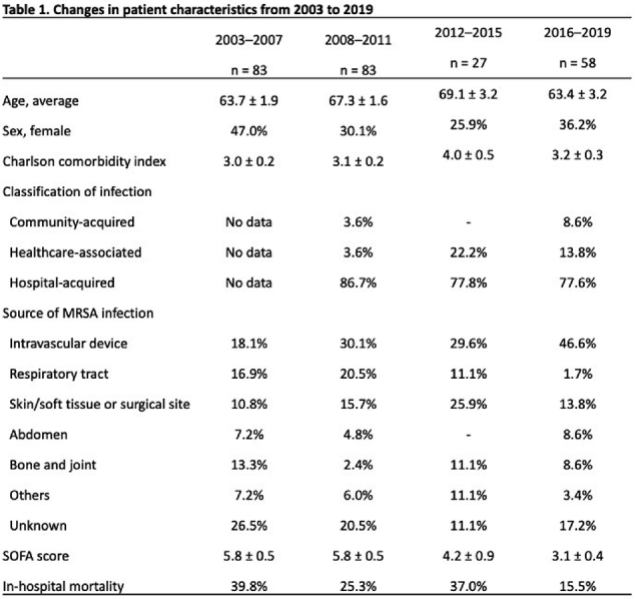

**Results:**

Between 2003 and 2019, SCCmec type II decreased from 79.2% to 15.5%, whereas type IV increased from 18.2% to 65.5%. Severity and outcomes also changed: SOFA score decreased from 5.8 to 3.1, while in-hospital mortality decreased from 39.8% to 15.5%. No changes in the patient background, such as age, sex, or underlying diseases, were observed (Table 1). Further, the percentage of intravascular device-related BSI increased significantly from 18.1% during 2003–2007 to 46.6% during 2016–2019.

The most common SCCmec type IV detected between 2012 and 2019 were ST8-IV and CC1-IV (Fig. 1A). ST8 was the predominant ST in both 2012–2015 and 2016–2019, but CC1 was detected only during 2016–2019 (Fig. 1B). In ST8-IV, MRSA/J and ST8-IV with spa type t5071 were identified, with MRSA/J being predominant. The detected MRSA/J, ST8-IV with spa type t5071, and CC1-IV subtypes were similar in terms of drug resistance, molecular characteristics, and phylogenetic features to those detected in the nationwide surveillance (Fig. 2). Patient characteristics across major MRSA types (ST8-IV, CC1-IV, ST8-I, ST5-II) revealed no significant differences, with lower SOFA scores and in-hospital mortality for CC1-IV (Table 2).

**Figure:**
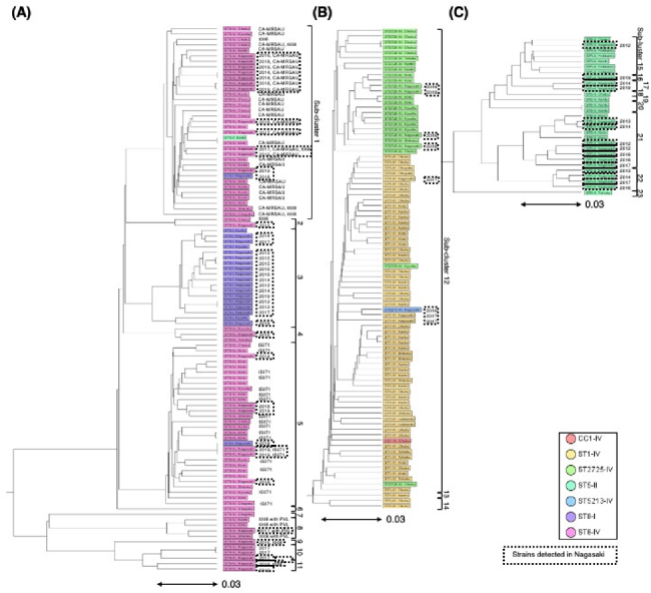

**Conclusion:**

The evolving nature of MRSA types in bloodstream infections, correlating with improved outcomes over time and influenced by changes in nationally and regionally circulating strains, is highlighted.

**Figure:**
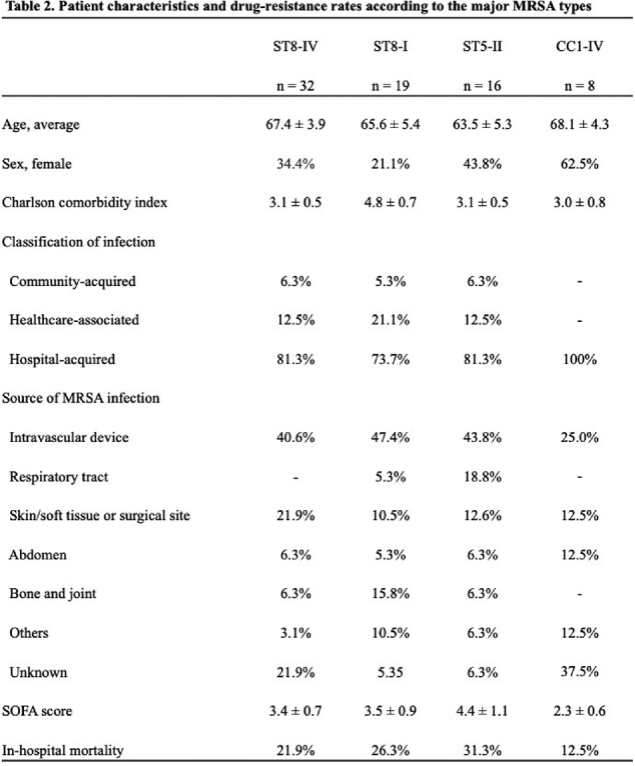

**Disclosures:**

**Hiroshi Mukae, M.D., Ph.D.**, AstraZeneca: Lecture fees|Gilead Sciences: Lecture fees|GSK: Lecture fees|MSD: Advisor/Consultant|MSD: Lecture fees|Pfizer: Lecture fees|Shionogi & Co., Ltd.: Advisor/Consultant|Shionogi & Co., Ltd.: Grant/Research Support|Shionogi & Co., Ltd.: Lecture fees

